# Eating and feeding behaviours in children in low‐income areas in Nairobi, Kenya

**DOI:** 10.1111/mcn.13023

**Published:** 2020-05-31

**Authors:** Antonina N. Mutoro, Ada L. Garcia, Elizabeth W. Kimani‐Murage, Charlotte M. Wright

**Affiliations:** ^1^ Human Nutrition, School of Medicine, Dentistry and Nursing, College of Medical, Veterinary & Life Sciences University of Glasgow Glasgow UK; ^2^ Maternal and Child Well‐being Unit African Population and Health Research Center Nairobi Kenya; ^3^ Child Health, School of Medicine University of Glasgow Glasgow UK

**Keywords:** appetite, children, force‐feeding, infant and young child feeding (IYCF), Kenya, malnutrition, responsive feeding

## Abstract

Child eating and caregiver feeding behaviours are critical determinants of food intake, but they are poorly characterized in undernourished children. We aimed to describe how appetite, food refusal and force‐feeding vary between undernourished and healthy children aged 6–24 months in Nairobi and identify potential variables for use in a child eating behaviour scale for international use. This cross‐sectional study was conducted in seven clinics in low‐income areas of Nairobi. Healthy and undernourished children were quota sampled to recruit equal numbers of undernourished children (weight for age [WAZ] or weight for length [WLZ] *Z* scores ≤2SD) and healthy children (WAZ > 2SD). Using a structured interview schedule, questions reflecting child appetite, food refusal and caregiver feeding behaviours were rated using a 5‐point scale. Food refusal and force‐feeding variables were then combined to form scores and categorized into low, medium and high. In total, 407 child–caregiver pairs, aged median [interquartile range] 9.98 months [8.7 to 14.1], were recruited of whom 55% were undernourished. Undernourished children were less likely to ‘love food’ (undernourished 78%; healthy 90% *p* = < 0.001) and more likely to have high food refusal (18% vs. 3.3% *p* = <0.001), while their caregivers were more likely to use high force‐feeding (28% vs. 16% *p* = 0.03). Undernourished children in low‐income areas in Nairobi are harder to feed than healthy children, and force‐feeding is used widely. A range of discriminating variables could be used to measure child eating behaviour and assess the impact of interventions.

**Key messages**

- Undernourished children were half as likely to ‘love food’ and six times more likely to have high food refusal, than healthy children. This is likely to have a negative impact on the treatment of undernutrition. A better understanding of the causes of food refusal is therefore required in order to effectively address this problem.
- Force‐feeding was used by around half of mothers, even where the child was well nourished. There is therefore a need for behaviour change interventions, which promote responsive feeding among caregivers.
- Better measures of child eating and caregiver feeding behaviour are required in low‐ and middle‐income countries.

## 1 INTRODUCTION

Eating behaviour is ‘the essential link between energy needs and energy intake’ because a child must eat in order to grow and thrive (Parkinson & Drewett, 2001). It has long been recognized that children with severe acute malnutrition (SAM) are often anorexic, but this is assumed to be a consequence of their nutritional state as appetite usually returns as refeeding proceeds (World Health Organization [WHO], 1999). In contrast, moderate acute malnutrition (MAM) is assumed to reflect insufficient food availability or diversity, and most treatment and prevention interventions have relied on the provision of fortified supplementary food (WHO, 2012). However, these have generally, shown only modest effects (Lazzerini, Rubert, & Pani, 2013), which is often attributed to noncompliance by families (Maleta et al., 2004), rather than the possibility that poor child appetite may influence child intake and in turn compliance (Lazzerini et al., 2013; Maleta et al., 2004).

There has been little research into how infants and young children eat in low‐ and middle‐income countries (LMICs), especially those with undernutrition, or how their caregivers respond to their eating behaviour (Abebe, Haki, & Baye, 2017; Moore, Akhter, & Aboud, 2006). Research in more affluent settings has found that infants with weight faltering, a form of MAM, have lower appetite and higher food aversion, suggesting that food refusal may also be a factor in the causation of undernutrition (Wright, Parkinson, & Drewett, 2006). The word ‘appetite’ means different things at different ages (Wright et al., 2006) and tends not to have an equivalent term in languages other than English, but other descriptions have been found to correlate with weight gain (Wright, Cox, & Le Couteur, 2011).

Therefore, we aimed to use a range of child and caregiver behaviours to create scores to identify low appetite, food refusal and force‐feeding in children aged 6–24 months in Nairobi and then test the extent to which these behaviours are associated with undernutrition.

## 2 METHODS

### 2.1 Study design and setting

This cross‐sectional study was conducted in seven of the 80 health facilities, which offer child welfare services and outpatient treatment for undernutrition in Nairobi. All were located in or on the periphery of major slums, where undernutrition remains a major public health problem, (Abuya, Ciera, & Kimani‐Murage, 2012; Kimani‐Murage et al., 2015). Five facilities, Mbagathi District Hospital, Kayole II Sub County Hospital, Makadara, Embakasi and Mukuru kwa Njenga Health Centre, were government run and two, Ruben Medical Clinic, Soweto PhC clinic, were faith‐based.

### 2.2 Sampling, inclusion and exclusion criteria

Children aged 6–24 months were quota sampled based on the severity of their nutritional status and whether they had started treatment with ready to use therapeutic foods. Undernourished children were eligible if they had weight for age (WAZ) or weight for length (WLZ) *Z* scores ≤ − 2SD. Any child with WAZ and or WLZ < −3SD was defined as SAM, with the remainder defined as MAM. Severely stunted children (<−3SD LAZ) and wasted (<−3SD WLZ) and moderately stunted (LAZ between −2SD and −3SD) children were classified as undernourished, as captured with a low WAZ. However, children who had low height (<−2SD LAZ) but higher weight (WAZ > −2SD) were classified as healthy. Children were excluded if they either had medical complications such as edema (*n* = 2), other medical conditions such as congenital heart disease (*n* = 1) or cleft lip and palate (*n* = 1). Undernourished children were recruited between February and July 2015, with an aim to include 150 children each with moderate versus severe undernutrition and 150 on treatment and 150 not on treatment. All eligible children identified in each heath facility were included until the quota for their subgroup was fulfilled.

The healthy children were recruited in a second round of data collection between July and August 2016 and were eligible if they had WAZ > −2 SD, using gender specific WHO growth charts. Healthy children were to be excluded if they had medical conditions, which required specialized care, but this did not arise in practice.

### 2.3 Research tools

Questions used to assess eating and feeding behaviours were developed, drawing on questions used in the Gateshead Millennium Study (GMS), a UK cohort study (Wright et al., 2006) supplemented by relevant questions from the Child Eating Behaviour Questionnaire (Wardle, Guthrie, Sanderson, & Rapoport, 2001) as well as behaviours observed during preliminary meal observations in low‐income areas in Nairobi (Mutoro, Garcia, & Wright, 2019).

Descriptions of all the items tested are shown in Tables S1–S4. Eating behaviour was assessed using nine variables, three hypothesized to relate to appetite and avidity and six to food refusal. The variables easy to feed, loves food and easily satisfied were used to assess appetite because we hypothesized that a child who is easy to feed and loves food is likely to have good appetite. The food refusal variables, spits out food, turns head away and holds food in mouth were selected because they have previously been shown to be associated with failure to thrive (Wilensky et al., 1996) and with slow weight gain in the GMS (Wright et al., 2006). Other refusal variables were included based on meal observations carried out by our group in similar settings in Nairobi (Mutoro et al., 2019).

Caregiver behaviour during meals was assessed using eight behaviours, of which four represented coercion or force‐feeding. The force‐feeding behaviours were selected based on meal observations in Kenya. Laissez faire feeding is relatively common LMICs (Dettwyler, 1989; Moore et al., 2006); to assess this, caregivers were asked how often they left their child alone when they refused to eat. There were also two questions about stress and anxiety related to feeding taken from the GMS (Wright et al., 2006) and two about whether children fed themselves during meals and snacks. Self‐feeding was assessed because studies show that self‐feeding is associated with increased food acceptance (Moore et al., 2006).

These were used to construct a structured interview schedule to be administered in Swahili, after forward and back translation to ensure accurate translation. All responses were coded using a 5‐point Likert scale, which ranged from 1 (*all the time*) to 5 (*not at all*). Data were collected by the researcher and five trained research assistants. Interviews lasted between 20 and 30 min and where possible were carried out in secluded areas of the health centres.

### 2.4 Anthropometry

The research team aimed to take all anthropometric measurements themselves using standardized equipment, but because of lack of space in the health facilities, in most cases, the anthropometric equipment available at the health facilities were used, but the research team assisted in taking measurements to standardize the techniques used (Lohman, Roache, & Martorell, 1992; WHO, 2008). Weight was measured using a digital weighing scale (SECA 385 digital weighing Scale III) to the nearest 0.1 kg where possible. Supine length was measured to the nearest 0.1 cm using a portable Rollameter (Raven Equipment Ltd., Dunmow, UK) or a UNICEF length board. Mid‐upper arm circumference (MUAC) was measured using MUAC tapes (S0145620 MUAC, Child 11.5 Red/PAC‐50) placed on the left arm at the midpoint between the elbow and shoulder recorded to nearest 0.1 cm.

### 2.5 Sample size

We planned to examine a wider range of novel behavioural and dietary variables between three subgroups and findings from preliminary meal observations suggested large differences in interest in food between healthy and undernourished children and in the proportion becoming upset during meals (Mutoro et al., 2019). We therefore aimed for a sample size sufficient (80% power, alpha 0.05) to detect a prevalence of 15% for any behaviour in one group compared with 30% in another (relative risk = 2). This required 150 subjects in each of the three subgroups.

### 2.6 Analysis

Analyses were conducted using Statistical Package for the Social Science (SPSS) IBM Corp. Released 2010 Version 19.0. Armonk, NY: IBM Corp and Epiinfo 7.1.5.2 Statcalc. Weight and length measurements were converted into *Z* scores using the WHO Anthro software version 3.2.2. Children were further classified as wasted, stunted, wasted and stunted if they had WLZ or LAZ ≤ −2SD or WLZ and LAZ ≤ −2SD, respectively. Spearman's (nonparametric) correlations were used to assess the strength and direction of interrelationships between individual child and caregiver variables and with WAZ scores, as a composite summary of the degree of stunting and/or wasting. Cronbach's alpha was used to assess internal consistency of variables. Variables, which showed reasonable consistency were then combined to create scores, a method used in previous studies (Bentley, Stallings, Fukumoto, & Elder, 1991, Gittelsohn et al., 1998, Wright et al., 2006). Where individual variables were used, the 5‐point Likert scale was recoded into three categories: (a) *all or most of the time* (1 & 2); (b) *sometimes* (3); and, (c) *rarely or never* (4 & 5).

Food refusal and force‐feeding scores were created by first subtracting each variable in the score with six to get an inverted value where by high scores reflected high frequency of occurrence. The mean of food refusal and force‐feeding variables was then calculated. Indices were used to assess the degree of interest in food, food refusal, force‐feeding and maternal anxiety. This was based on the assumption that children and caregivers were likely to experience these behaviours at one point during meals, but only the frequency of occurrence and the number of behaviours during meals are a likely indicator of extreme behaviour (Dettwyler, 1989). The mean of behaviours was then used to create categories reflecting high, moderate and low occurrence.

Logistic regression was used to test the association between eating and feeding behaviour indices and nutritional status (healthy vs. undernourished). All children were included in descriptive analysis, but when creating scores, children with missing data were excluded.

### 2.7 Ethical considerations

Ethics approval for the study was obtained from the University of Glasgow Ethics review committee (200140057), University of Nairobi and Kenyatta National Hospital Ethics Review committee (P651/11/2014) and the National Council for Science, Technology and Innovation (NACOSTI/P/15/9164/5185). Access to health facilities was granted by the Nairobi county and subcounty health offices.

## 3 RESULTS

**FIGURE 1:**
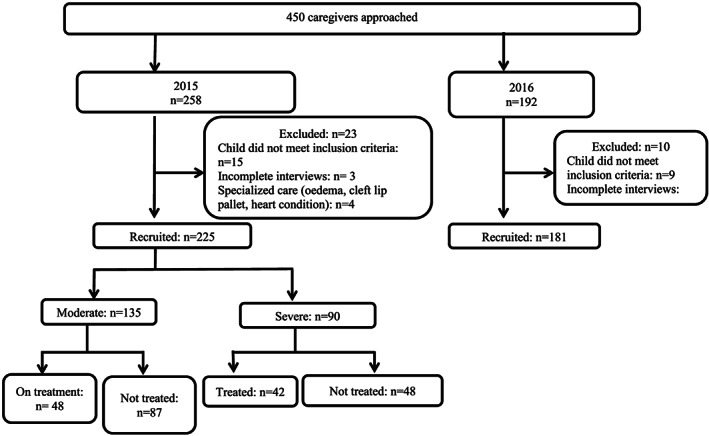
Flow chart showing participant recruitment

In total, 450 caregivers were approached for interviews, 415 were interviewed, but only 407 were included in the analysis (Figure 1). We were able to recruit 182 healthy children, but only 135 (23%) MAM and 90 (22%) SAM children. Child and caregiver characteristics and the growth data of the children are presented in Table 1. Within the undernourished children, 69% (155) were wasted (WLZ < −2SD), 28% (114) were stunted and 24% (55) had MUAC <11.5 cm and only 20% (46) had MUAC above 12.5 cm. Over half of the caregivers reported encouraging children during meals. Reported child self‐feeding was not common: only 4% children were reported to feed themselves during main meals and 33% during snacks. Healthy (39%) and older (57%) children were more likely to feed themselves snacks than undernourished (28%; *p* = 0.013) and young children (16%; *p* < 0.001). Compared with children who fed themselves snacks, children who did not were two times more likely to be undernourished, odds ratio [95% confidence interval] 2.23[1.36, 3.67; *p* = 0.002]. This association remained significant after adjusting for child age. However, no association between nutritional status, child age and self‐feeding of meals was observed.

**TABLE 1:** Child and caregiver characteristics (values are *n* (%) unless otherwise stated)

Child characteristics	All	Nutrition status	Undernourished	Severity	SAM	Treatment status	Not treated
		Healthy		MAM		On treatment	
	407	182 (44.7)	225 (55.3)	135 (33.2)	90 (22.1)	90 (40.0)	135 (60.0)
Female	221 (54.3)	93 (42.1)	128 (57.9)	85 (38.5)	43 (19.5)	128 (43.8)	72 (56.3)
Age in months^a^	9.98 [8.7 to 14]	9.72 [8.5 to 13.0]	10.2 [8.9 to 15.0]	9.98 [8.7 to 14.0]	10.7 [9.0 to 16.0]	11.1 [9.03 to 15.0]	9.99 [8.6 to 14.0]
Weight for age *Z* scores^a^	−2.07 [−2.9 to −0.5]	−0.38 [−0.8 to 0.5]	−2.82 [−3.3 to −2.3]	−2.43 [−2.7 to −2.2]	−3.53 [−3.8 to −3.2]	−2.94 [−3.5 to −2.5]*	−2.74 [−3.2 to −2.2]
Weight for length *Z* scores^a^	−2.40 [−1.5 to −0.1]	0.14 [−0.6 to 0.8]	−2.32 [−2.9 to −1.8]	−2.19 [−2.6 to −1.7]	−2.78[−3.4 to −2.2]	−2.56 [−3.3 to −1.8]	−2.29 [−2.7 to −1.8]
Length for age *Z* scores^a^	−1.32 [−2.3 to −0.4]	−0.54 [−1.2 to 0.3]	−2.05 [−2.9 to −1.3]	−1.72[−2.3 to −1.0]	−2.76 [−3.4 to −2.2]	−2.94 [−3.5 to −2.5]	−1.95 [−2.7 to −1.3]
**Family characteristics**							
Caregiver's age in years^a^	26.0 [22 to 29]	25 [22 to 29]	26.0 [23 to 29]	26.0 [22 to 29]	26.0 [23 to 29]	27.0 [23 to 30.0]	25.0 [22 to 29]
*p* value		0.11		0.11		0.17	
**Number of children under 5 years**							
One child	310 (76.5)	150 (83.3)	160 (71.1)	96 (71.1)	64 (71.1)	61 (67.8)	99 (73.3)
More than one child	95 (23.5)	30 (16.7)	65 (28.9)	39 (28.9)	26 (28.9)	29 (32.2)	36 (26.7)
*p* value		0.01		0.01		0.37	
**Caregiver's education**							
Primary and below	162 (39.9)	119 (65.4)	125 (55.8)	73 (54.1)	52 (58.4)	48 (53.9)	77 (57.0)
Secondary and above	244 (60.1)	63 (34.6)	99 (44.2)	62 (45.9)	37 (41.6)	41 (46.1)	58 (43.0)
*p* value		0.05		0.15		0.65	
**Housing**							
Semipermanent	171 (42.0)	78 (42.9)	93 (41.3)	51 (37.8)	42 (46.7)	34 (37.8)	59 (43.7)
Permanent	236 (58.0)	104 (57.1)	132 (58.7)	84 (62.2)	48 (53.3)	56 (62.2)	76 (56.3)
*p* value		0.76		0.74		0.38	

Abbreviations: MAM, moderate acute malnutrition; SAM, severe acute malnutrition.

^a^ Median [interquartile range].

The occurrence of individual child eating and caregiver feeding behaviours and their intercorrelations are presented in Tables S1–S4. Two of the three hypothesized appetite variables (‘easy to feed’ and ‘easily satisfied’) showed weak and inconsistent correlations with each other and with WAZ, so ‘loves food’ was used as a single appetite‐related variable, as it was correlated with weight gain and had face validity. The five food refusal variables, turns away from food, spits out food, cries during meals, holds food in mouth and pushes food away, were all significantly intercorrelated (internal consistency, Cronbach alpha =0.72) and significantly correlated with WAZ (Table S2). The inverted mean of these five variables was thus used to form a food refusal score (Table 3). The maternal feeding behaviours on the whole showed weak intercorrelation, but three force‐feeding variables (restrains child, pours food in child's mouth and forces mouth open) were moderately and significantly intercorrelated and two of these (restrains child and forces mouth open) were significantly inversely related to WAZ. The inverted mean of these three variables was thus used to form a force‐feeding score (see Table 3). The two maternal stress/anxiety variables, worries child not eating enough and finds feeding stressful, were moderately correlated with each other and both significantly inversely correlated with WAZ, but were felt to be too few to combine as a score and were thus used as two individual variables. All the resulting scores and items were significantly intercorrelated, and most were also significantly correlated with WAZ (Table 2).

**TABLE 2:** Eating and feeding behaviour score categories for total sample values are (Spearman's *r*)

Behaviour	*n* (%)	Food refusal	Force‐feeding	Finds feeding stressful	Worry child not eating enough	Weight for age *Z* score	Age
**Loves food** (single variable)							
All the time	230 (56.5)	**−0.38**	**−0.34**	**−0.31**	**−0.29**	**−**0.11^*^	−0.05
Sometimes	110 (27.0)	—	—	—	—	—	—
Not at all	67 (16.5)	—	—	—	—	—	—
**Food refusal** Mean of 5 food refusal variables (inverted): Turns head away, spits out food, cries during meals, holds food in mouth, pushes food away							
High (4–5)	46 (11.3)	—	**0.42**	**0.44**	**0.54**	**−0.31**	0.03
Moderate (3–4)	114 (28.9)	—	—	—	—	—	—
Low (1–2)	247 (60.7)	—	—	—	—	—	—
**Force‐feeding** Mean of 3 food refusal variables (inverted): Restrains child, pours food in child's mouth, forces mouth open							
High (3–5)	90 (22.7)	—	—	**0.23**	**0.29**	**−0.14**	0.07
Moderate (2)	121(30.6)	—	—	—	—	—	—
Low (1)	185 (46.7)	—	—	—	—	—	—
**Finds feeding stressful** (single variable)							
All the time	89 (21.9)	—	—	—	**0.48**	**−0.22**	0.04
Sometimes	85 (20.9)	—	—	—	—	—	—
Not at all	232 (57.1)	—	—	—	—	—	—
**Worry child not eating enough** (single variable)							
All the time	134 (33.0)	—	—	—	—	**−0.47**	0.05
Sometimes	118 (29.1)	—	—	—	—	—	—
Not at all	154 (37.9)	—	—	—	—	—	—

*Note*: Intercorrelation (Spearman's *r*; all values in bold are statistically significant *p* < 0.01 except where marked ^*^ *p* = 0.05; other unbolded values were *p* > 0.05).

^*^ *p* = 0.05.

Compared with healthy children, undernourished children were less likely to love food, have high food refusal and their caregivers were more likely to be anxious about their eating habits. There was no difference between those with SAM and MAM, except that force‐feeding was significantly more common in the children with SAM (Table 3). Undernourished children showed similar eating behaviour, whether on or off treatment, but mothers of undernourished children not on treatment were significantly more likely to find feeding stressful and to worry that their child was not getting enough to eat.

**TABLE 3:** Association between nutritional status and its treatment and child eating and caregiver feeding behaviour

Behaviour	Nutrition status		Severity		Treatment status	
	Healthy (*n* = 182)	Undernourished (*n* = 225)	MAM (*n* = 135)	SAM (*n* = 90)	Undernourished on treatment (*n* = 90)	Undernourished not treated (*n* = 135)
	*n* (%)	*n* (%)	*n* (%)	*n* (%)	*n* (%)	*n* (%)
**Loves food**						
All the time	117 (64.3)	113 (50.2)	73 (54.1)	40 (44.4)	72 (53.3)	41 (45.6)
Sometimes	47 (25.8)	63 (28.0)	36 (26.7)	27 (30.0)	36 (26.7)	27 (30.0)
Rarely	18 (9.9)	49 (21.8)	26 (19.3)	23 (25.6)	27 (20.0)	22 (24.4)
*p χ* ^2^ linear trend	<0.001^a^		0.12^b^		0.23^c^	
**Food refusal**						
High	6 (3.3)	40 (17.8)	18 (13.3)	22 (24.4)	16 (17.8)	24 (17.8)
Moderate	36 (19.8)	78 (34.7)	41 (45.6)	27 (30.0)	30 (33.3)	48 (35.6)
Low	140 (79.6)	107 (47.6)	66 (48.9)	41 (45.6)	44 (48.9)	63 (46.7)
*p χ* ^2^ linear trend	<0.001^a^		0.12^b^		0.89^c^	
**Force‐feeding**						
High	29 (16.0)	61 (28.4)	35 (27.1)	38 (44.2)	19 (22.9)	42 (31.8)
Moderate	62 (34.3)	59 (27.4)	37 (28.7)	22 (25.6)	29 (34.9)	30 (22.7)
Low	90 (49.7)	95 (44.4)	57 (44.2)	26 (30.2)	35 (42.2)	60 (45.5)
*p χ* ^2^ linear trend	0.03^a^		0.011^b^		0.69^c^	
**Finds feeding stressful**						
High	26 (14.3)	63 (28.1)	33 (24.6)	30 (33.3)	25 (27.8)	63 (47.0)
Moderate	35 (19.2)	50 (22.3)	31 (23.1)	19 (21.1)	17 (18.9)	33 (24.6)
Low	121 (66.5)	111(49.6)	70 (52.2)	41 (45.6)	48 (53.3)	38 (28.4)
*p χ* ^2^ linear trend	<0.001^a^		0.22^b^		<0.001^c^	
**Worry child does not eat enough**						
High	26 (14.3)	108 (48.2)	64 (47.8)	44 (48.9)	33 (36.7)	75(56.0)
Moderate	41 (22.5)	77 (34.4)	41 (30.6)	36 (40.0)	38 (42.2)	39 (29.1)
Low	115 (63.2)	39 (17.4)	29 (21.6)	10 (11.1)	19 (21.1)	20 (14.9)
*p χ* ^2^ linear trend	<0.001^a^		0.30^b^		0.02^c^	

Abbreviations: MAM, moderate acute malnutrition; SAM, severe acute malnutrition.

^a^ Healthy versus undernourished.

^b^ MAM versus SAM.

^c^ Undernourished on versus off treatment.

There was no association between eating behaviour and gender or child's age and no significant difference in eating behaviour between wasted or stunted versus wasted children (results not shown). Logistic regression analysis assessing the association between eating and feeding behaviour and nutritional status are presented in full in Table S5. All eating and feeding behaviour variables were associated with nutritional status in the univariate analysis, but when all were included in the same model, only food refusal and worry that child does not eat enough were significantly associated with nutritional status and severity. Compared with children with low food refusal, children with high food refusal had high odds of being undernourished odds ratio [confidence interval; *p* value] 4.45 [1.6, 13.0; *p* = 0.006].

Force‐feeding was significantly more common in undernourished children in univariate analysis, but it did not prove to be an independent predictor of nutritional status in the logistic regression model. Force‐feeding correlated strongly with all the other eating and feeding measures but the only independent predictors of high force‐feeding were liking for food and food refusal (Table 4).

**TABLE 4:** Logistic regression assessing the predictors of low versus high force‐feeding

	Univariate		Adjusted	
Predictor (reference)	Odds ratio [95% confidence interval]	*p* value	Odds ratio [95% confidence interval]	*p* value
**Loves food (all the time)**				
Sometimes	1.52 [0.96, 2.43]	0.08	1.34 [0.81, 2.19]	0.25
Not at all	8.27 [3.91, 17.5]	<0.001	5.63 [2.51, 12.6]	<0.001
**Food refusal (low)**				
Medium	4.03 [2.45, 6.61]	<0.001	3.02 [1.72, 5.32]	<0.001
High	2.85 [1.46, 5.57]	0.01	1.28 [0.54, 3.01]	0.57
**Worry child does not eat enough (low)**				
Medium	2.91 [1.78, 4.73]	<0.001		
High	1.63 [1.00, 2.67]	0.05		
**Stress feeding (low)**				
Medium	1.23 [0.75, 2.03]	0.42		
High	3.56 [2.05, 6.25]	<0.001		
**Nutrition status (healthy)**	1.25 [0.84, 1.85]	0.27		

Adjusted: mutual adjustment for eating and feeding behaviours and nutrition status.

## 4 DISCUSSION

In this study, we aimed to create scores to identify low appetite, food refusal and force‐feeding in children aged 6–24 months in Nairobi and then describe the extent to which these behaviours were associated with undernutrition. Eating and feeding behaviour in this study was assessed using questions adapted from an earlier UK questionnaire (Wright et al., 2006). While a number of questions proved relevant to food refusal and force‐feeding, few of the questions were potentially relevant to appetite, and only one proved useable. However, this variable revealed that two thirds of healthy children ‘loved food’ all the time, while less than a quarter showed food refusal. In contrast, undernourished children were less likely to love food and six times more likely to have high food refusal. Force‐feeding was relatively common in both groups and was more likely to happen in those with least liking for food, regardless of their state of nutrition.

These findings are consistent with the few studies of child eating behaviour conducted in other LMICs (Abebe et al., 2017; Dettwyler, 1989; Moore et al., 2006). In rural Ethiopia, a meal observation study found that stunted children aged 12–23 months accepted only half as many mouthfuls of food as healthy children (Abebe et al., 2017). Similarly, in rural Bangladesh, Moore and colleagues found that moderately undernourished children aged 8–24 months were less likely to complete meals (Moore et al., 2006). It is, however, not clear if the low interest in food found here is a cause or an effect on undernutrition. In SAM, low interest in food may reflect the undernourished state itself, where the body undergoes reductive adaptation to conserve energy and maintain vital body functions, but this would be expected to resolve once the child was on treatment. However, in this cohort, there was no difference in eating behaviour between those with severe and moderate malnutrition, or between those on and off treatment. Low interest in food may also be explained by recurrent infections or micronutrient deficiencies, which are prevalent in low‐income areas (African Population and Health Research Center, 2014; Ferguson, Chege, Kimiywe, Wiesmann, & Hotz, 2015; Mberu, Haregu, KYobutungi, & Ezeh, 2016) or simply reflect an intrinsically low drive to eat, the antithesis of the hungry obese prone child, that could be genetic in origin (Wardle & Carnell, 2009).

Force‐feeding was relatively common in this study, but seems to be neither a risk factor for or protective against undernutrition. Force‐feeding, often characterized by threats and physical restraint, has previously been reported in LMICs (Abebe et al., 2017, Bentley et al., 1991, Ha et al., 2002, Moore et al., 2006, Nti & Lartey, 2007, Oni et al., 1991). Although force‐feeding is not an ideal feeding method, it has been associated with better food acceptance in some children (Ha et al., 2002) and thus the use of force‐feeding could even protect some children with food refusal against becoming undernourished; conversely, the use of force‐feeding may cause aversive responses and food refusal. This illustrates the complexity of measuring EFB cross‐sectionally, where there are strong correlations between different dimensions, leading to multicollinearity (Tu, Kellett, Clerehugh, & Gilthorpe, 2005) and no single direction of causation. For every action by the child, there is a maternal reaction and vice versa. This can only be disentangled in future by prospective research designs or, ideally, intervention studies.

Children were generally not given opportunities to feed themselves, especially during meals, which appears to be a common characteristic of meals in LMIC (Abebe et al., 2017; Moore et al., 2006), even though child self‐feeding is associated with higher food acceptance (Dearden et al., 2009; Moore et al., 2006). A study in rural Bangladesh, observed that only a quarter of moderately undernourished young children were allowed to feed themselves, despite having the ability and interest to do so (Moore et al., 2006). This may reflect the fact that meals were mainly mashed, rather than finger foods. Parental feeding of children may be a sign of limited time available for child care (Moore et al., 2006) as well as avoidance of food wastage, especially in food insecure households (Affleck & Pelto, 2012). Some children may be unable to feed themselves due to delayed motor development, especially among undernourished children, but this was not assessed in the current study.

Eating and feeding behaviour in this study was assessed using a set of questions adopted from a questionnaire that was initially designed and tested in the United Kingdom (Wright et al., 2006). Caregivers were able to understand and respond to these questions when translated to Swahili, but it is possible that cultural, linguistic and functional equivalence of some of the questions were not achieved. Specifically, few of the questions used reflected appetite and maternal anxiety, so more studies are required to expand the interview schedule and test its validity and reliability in other languages in LMICs.

Ideally, meal observations in addition to interviews could have been done to validate caregiver reports, which may not be reliable. For example, most caregivers reported encouraging their children when they refused to eat, but our observation studies in a similar setting (Mutoro et al., 2019) and in other LMICs (Bentley, Stallings, et al., 1991, Engle & Zeitlin, 1996, Moore et al., 2006, Mutoro, 2018) suggest that caregivers are usually passive during meals. However, conducting meal observations in this setting has already proved difficult (Mutoro et al., 2019) and may not reflect typical, habitual behaviour. Other survey‐based studies have demonstrated that parental reports are generally valid. A study in Peru showed a significant decrease in energy intake during periods when caregivers reported poor appetite in their children (Brown, Peerson, Lopez DE Romana, Kanashiro, & H. C., & Black, R. E., 1995). Another study in rural Bangladesh found that 85% of caregivers who reported feeding problems experienced at least one food refusal during meal observations (Moore et al., 2006). In this case, the multiple measures we used of food refusal enabled the ‘quantification’ of how often behaviours occurred and thus the extent to which these might be problematical. Future work is needed to find a range of behaviours that can similarly be used to quantify appetite.

Because of the quota sampling approach, the sample recruited were not representative of the general population, but the inclusion of a large sample of undernourished children allowed us to detect differences in eating and feeding behaviour between healthy and undernourished children that would not be detectable in a population‐based study, with proportionally fewer undernourished children. The definition of undernutrition we used identified a mixture of stunted and wasted children, with wasting being commonest. We included SAM children because we also wanted to assess the impact of ready to use foods on eating and feeding behaviour, and most children receiving these were SAM. However, we did not assess how long children had been on treatment, which is a limitation of this study. We also wanted to consider the extent to which children with SAM differed from those who were MAM. It is of interest that the MAM and SAM children were generally more similar to each other rather than to the well‐nourished children.

The healthy and undernourished children were recruited a year apart, so it is possible that time‐dependent factors may have produced differences, but the environmental conditions in the slums were the same, so it is likely that all surveyed children were exposed to the same conditions.

### 4.1 Implications

This study highlights the need for a better recognition that simply providing more food may not be enough to ensure better intake in young undernourished children. Responsive feeding intervention studies have already shown that it is possible to modify eating and feeding behaviour in healthy children (Aboud & Akhter, 2011; Aboud, Shafique, & Akhter, 2009; Vazir et al., 2013). These findings suggest that to ensure successful rehabilitation of undernourished children, behaviour change interventions may be needed to identify food refusal and help caregivers to respond appropriately.

## 5 CONCLUSION

We have identified groups of behaviours that suggest food refusal and force‐feeding, but only a limited description of enthusiasm for eating in a low‐income urban area in Nairobi, Kenya. These suggest that undernourished children have less liking for food and much higher food refusal than their healthy peers. Force‐feeding was also common, but was not clearly related to nutritional status. A high prevalence of food refusal has important implications for successful treatment of malnutrition.

## CONFLICTS OF INTEREST

The authors declare that they have no conflicts of interest.

## CONTRIBUTIONS

CMW and ANM designed the research. ANM collected the data. ANM and CMW analysed the data. ANM wrote the first draft. ANM, CMW, ALG and EWK reviewed the subsequent drafts. ANM had primary responsibility for the final content. All authors have read and approved the final manuscript.

## Supporting information

Table S1: Inter‐correlations between appetite/avidity questions
Table S2: Inter‐correlations between food refusal questions
Table S3: Intercorrelations between maternal feeding behaviours
Table S4: Maternal feeding stress
Table S5: Logistic regression analysis assessing the association between eating and feeding behaviours and nutrition status (healthy vs undernourished)
