# Supplementary material for: Eating and feeding behaviours in children in low‐income areas in Nairobi, Kenya
Source: Matern Child Nutr. 2020 May 31;16(4):e13023. doi: 10.1111/mcn.13023 (PMC7506998; doi:10.1111/mcn.13023)
Supplement: Supplementary file 1 — Table S1: Inter‐correlations between appetite/avidity questions Table S2: Inter‐correlations between food refusal questions Table S3: Intercorrelations between maternal feeding behaviours Table S4: Maternal feeding stress Table S5: Logistic regression analysis assessing the association between eating and feeding behaviours and nutrition status (healthy vs undernourished) [file MCN-16-e13023-s001.docx]

**Supplementary Table 1: Inter-correlations between appetite/avidity questions**

|  |  | **Inter-correlations (Spearman’s R)** | | |
| --- | --- | --- | --- | --- |
| **Child behaviour** | n (%) all children | **Loves food** | **Easily satisfied** | **Weight for age** |
| **Easy to feed** |  |  |  |  |
| All/Most of the time | 250 (61.6) | **0.49** | -0.09 | 0.00 |
| Sometimes | 116 (28.6) |  |  |  |
| Rarely/Not at all | 40 (9.9) |  |  |  |
| **Loves food** |  |  |  |  |
| All/Most of the time | 230 (56.5) | - | -0.12 ^a^ | -0.11 ^a^ |
| Sometimes | 110 (27.0) |  |  |  |
| Rarely/Not at all | 67 (16.5) |  |  |  |
| **Easily satisfied** |  |  |  |  |
| All/Most of the time | 183 (45.0) | - | - | 0.09 ^a^ |
| Sometimes | 106 (26.0) |  |  |  |
| Rarely/Not at all | 118 (29.0) |  |  |  |

Likert scales: 1=all the time; 5= Not at all **Bold values**: P=.01 ^a^ P=.05

**Supplementary Table 2: Inter-correlations between food refusal questions**

|  |  | **Inter-correlation (Spearman’s R)** | | | | | |
| --- | --- | --- | --- | --- | --- | --- | --- |
| **Child behaviour** | **n (%) all children** | **Turns away** | **Pushes food away** | **Cries/ screams** | **Holds food in mouth** | **Spits out food** | **Weight for age Z scores** |
| **Eats slowly** |  | **0.30** | **0.33** | **0.28** | **0.21** | **0.22** | **0.19** |
| All/most of the time | 201 (49.4) |  |  |  |  |  |  |
| Sometimes | 140 (34.4) |  |  |  |  |  |  |
| Rarely/not at all | 66 (16.2) |  |  |  |  |  |  |
| **Turns away** |  | 1 | **0.49** | **0.44** | **0.21** | **0.33** | **0.24** |
| All/most of the time | 146 (35.9) |  |  |  |  |  |  |
| Sometimes | 183 (45.0) |  |  |  |  |  |  |
| Rarely/not at all | 78 (19.2) |  |  |  |  |  |  |
| **Pushes food away** |  |  | 1 | **0.38** | **0.18** | **0.35** | **0.28** |
| All/most of the time | 123 (30.2) |  |  |  |  |  |  |
| Sometimes | 160 (39.3) |  |  |  |  |  |  |
| Rarely/not at all | 124 (30.5) |  |  |  |  |  |  |
| **Cries/screams** |  |  |  | 1 | **0.22** | **0.24** | 0.12^a^ |
| All/most of the time | 86 (21.1) |  |  |  |  |  |  |
| Sometimes | 134 (32.9) |  |  |  |  |  |  |
| Rarely/not at all | 187 (45.9) |  |  |  |  |  |  |
| **Holds food in mouth** |  |  |  |  | 1 | **0.18** | **0.14** |
| All/most of the time | 64 (15.7) |  |  |  |  |  |  |
| Sometimes | 89 (21.9) |  |  |  |  |  |  |
| Rarely/not at all | 254 (62.4) |  |  |  |  |  |  |
| **Spits out food** |  |  |  |  |  | **1** | **0.16** |
| All/most of the time | 91 (22.4) |  |  |  |  |  |  |
| Sometimes | 170 (41.8) |  |  |  |  |  |  |
| Rarely/not at all | 146 (35.9) |  |  |  |  |  |  |

Likert scales: 1=all the time; 5= Not at all; **Bold values**: P=.01 ^a^ P=.05

**Supplementary Table 3: Intercorrelations between maternal feeding behaviours**

|  |  | **Inter-correlation (Spearman’s R)** | | | | | | | |
| --- | --- | --- | --- | --- | --- | --- | --- | --- | --- |
| **Child behaviour** | **n (%) all children** | **Offers something else** | **Leaves child alone** | **Restrains child** | **Pours food into mouth** | **Forces mouth open** | **Threatens child** | **Hold’s child’s nose** | **Weight for age Z scores** |
| **Encourages child** |  |  |  |  |  |  |  |  |  |
| All the time | 238 (58.5) | **0.17** | 0.1 | 0.04 | **-0.18** | -0.07 | 0.07 | 0.07 | -0.01 |
| Sometimes | 147 (33.4) |  |  |  |  |  |  |  |  |
| Rarely/Not at all | 33 (8.1) |  |  |  |  |  |  |  |  |
| **Offers something else** | |  |  |  |  |  |  |  |  |
| All the time | 164 (40.6) | 1 | -0.07 | 0.11 ^a^ | 0.04 | -0.02 | 0.04 | **0.03** | **0.16** |
| Sometimes | 147 (36.4) |  |  |  |  |  |  |  |  |
| Rarely/Not at all | 93 (23.0) |  |  |  |  |  |  |  |  |
| **Leaves child alone** |  |  |  |  |  |  |  |  |  |
| All the time | 66 (16.3) |  |  |  |  |  |  |  |  |
| Sometimes | 214 (52.8) |  | 1 | 0.08 | 0.03 | 0.05 | 0.09 | **0.19** | 0.01 |
| Rarely/Not at all | 125 (30.9) |  |  |  |  |  |  |  |  |
| **Restrains child** |  |  |  |  |  |  |  |  |  |
| All the time | 129 (31.7) |  |  | 1 | **0.37** | **0.39** | 0.03 | **0.06** | **0.14** |
| Sometimes | 116 (28.5) |  |  |  |  |  |  |  |  |
| Rarely/Not at all | 162 (39.8) |  |  |  |  |  |  |  |  |
| **Pours food into child’s mouth** | |  |  |  |  |  |  |  |  |
| All the time | 40 (10.1) |  |  |  | 1 | **0.44** | 0.01 | 0.01 | 0.08 |
| Sometimes | 99 (20.7) |  |  |  |  |  |  |  |  |
| Rarely/Not at all | 275 (69.3) |  |  |  |  |  |  |  |  |
| **Forcefully opens child’s mouth** | |  |  |  |  |  |  |  |  |
| All the time | 64 (15.8) |  |  |  |  | 1 | 0.05 | 0.08 | 0.12^a^ |
| Sometimes | 99 (24.4) |  |  |  |  |  |  |  |  |
| Rarely/Not at all | 243 (59.9) |  |  |  |  |  |  |  |  |
| **Threatens** |  |  |  |  |  |  |  |  |  |
| All the time | 10 (2.5) |  |  |  |  |  | 1 | **0.19** | **-0.25** |
| Sometimes | 30 (7.4) |  |  |  |  |  |  |  |  |
| Rarely/Not at all | 367 (90.2) |  |  |  |  |  |  |  |  |

Likert scales: 1=all the time; 5= Not at all; **Bold values**: P=.01; ^a^P=.05

**Supplementary Table 4:** **Maternal feeding stress**

|  |  | **Inter-correlation (Spearman’s R)** | |
| --- | --- | --- | --- |
|  | n (%) **all children** | **Worry child does not get enough to eat** | **Weight Z score** |
| **Stress feeding child** |  |  |  |
| All the time | 89 (21.9) | **0.47** | **0.22** |
| Sometimes | 85 (20.9) |  |  |
| Rarely/Not at all | 232 (57.1) |  |  |
| **Worry child does not eat enough** |  |  |  |
| All the time | 134 (33.0) |  | **0.47** |
| Sometimes | 118 (29.1) |  |  |
| Rarely/Not at all | 154 (37.9) |  |  |

Likert scales: 1=all the time; 5= Not at all; **Bold values**: P=.01 ^a^ P=.05

Supplemental Table 5: Logistic regression analysis assessing the association between eating and feeding behaviours and nutrition status (healthy vs undernourished)

|  | Univariate | | Adjusted for all other variables shown | |
| --- | --- | --- | --- | --- |
| **Predictor (reference)** | **Odds ratio [95% Confidence Interval]** | **P value** | **Odds ratio [95% Confidence Interval]** | **P value** |
| **Healthy vs undernourished** |  |  |  |  |
| **Loves food (all the time)** |  |  |  |  |
| Sometimes | 1.39 [0.88 to 2.19] | 0.160 |  |  |
| Not at all | 2.82 [1.54to 5.13] | 0.001 |  |  |
| **Food refusal (low)** |  |  |  |  |
| Medium | 2.84 [1.78 to 4.53] | <0.001 | 2.09 [1.15 to 3.70] | 0.015 |
| High | 8.72 [3.57 to 21.3] | <0.001 | 4.45 [1.62 to 13.0] | 0.006 |
| **Force-feeding (low)** |  |  |  |  |
| Medium | 0.90 [0.57 to 1.43] | 0.658 |  |  |
| High | 1.20 [1.18 to 3.34] | 0.010 |  |  |
| **Stress feeding (low)** |  |  |  |  |
| Medium | 1.56 [0.94 to 2.56] | 0.084 |  |  |
| High | 2.64 [1.56 to 4.46] | <0.001 |  |  |
| **Worry child does not eat enough (low)** |  |  |  |  |
| Medium | 5.54 [3.28 to 9.36] | <0.001 | 7.22 [3.96 to 13.1] | <0.001 |
| High | 12.3 [6.99 to 21.5] | <0.001 | 12.2 [6.03 to 24.5] | <0.001 |
